# The Senescence-SASP Landscape in Colon Adenocarcinoma: Prognostic and Therapeutic Implications

**DOI:** 10.3390/cimb48010114

**Published:** 2026-01-21

**Authors:** Tianyu Ren, Suyouwei Gao, Yangrong Feng, Yangyang Xu, Xinyi Mi, Jite Shi, Man Chu

**Affiliations:** Faulty of Medical Technology, Shaanxi University of Chinese Medicine, Xianyang 712046, China; 224070812899@email.sntcm.edu.cn (T.R.); 523070302450@email.sntcm.edu.cn (S.G.); 225070812891@email.sntcm.edu.cn (Y.F.); 2071030@sntcm.edu.cn (Y.X.); 522070302446@email.sntcm.edu.cn (X.M.); 2071042@sntcm.edu.cn (J.S.)

**Keywords:** colon adenocarcinoma, cellular senescence, SASP, prognosis

## Abstract

Cellular senescence, characterized by permanent cell cycle arrest, significantly influences cancer development, immune regulation, and progression. However, the precise mechanisms by which senescence contributes to colorectal cancer prognosis remain to be fully elucidated. By integrating expression profiles of senescence-related and prognostic genes in colon adenocarcinoma (COAD) patients, we formulated and confirmed a nine-gene cellular senescence-related signature (CSRS) that integrates senescence-associated and prognosis-predictive genes using data from the CellAge, The Cancer Genome Atlas (TCGA) and Gene Expression Omnibus (GEO). A cell senescence-related prognostic formula was developed as follows: CSRS = (CASP2 × 0.2098) + (CDKN2A × 0.1196) + (FOXD1 × 0.1472) + (ING5 × 0.3723) + (OXTR × 0.0786) + (PHGDH × 0.1408) + (SERPINE1 × 0.1127) + (SNAI1 × 0.1034) + (LIMK1 × 0.0747). In a multivariate Cox proportional hazards model, the CSRS score, age and TNM stage were all identified as significant independent indicators for overall survival, affirming their prognostic value in colorectal cancer. The CSRS-high group exhibited significantly up-regulated senescence-associated secretory phenotype (SASP) and immune cell infiltration, whereas the CSRS-low group showed an apparent better response to immune-checkpoint inhibitor therapy. Our findings suggest CSRS score and its constituent genes represent potential biomarkers for prognosis and immunotherapeutic benefit in COAD patients. Extending this nine-gene set into a broader senescence-associated panel should be a next step toward delivering truly individualized treatment plans.

## 1. Introduction

Colorectal cancer is the third most frequently occurring cancer and ranks second in causing cancer deaths globally [1]. Owing to low rates of early detection, the majority of patients are diagnosed with metastatic cancer which translates to a drastically reduced 5-year survival rate (below 20%) and a highly unfavorable prognosis [2]. Despite the utilization of current modalities including chemotherapy, molecular targeted therapy and immunotherapy, the improvement in survival outcomes for patients with advanced colorectal cancer remains modest [3]. Thus, there is an urgent need to implement risk-stratified, individualized treatment and to identify reliable prognostic biomarkers.

Cellular senescence functions as a pivotal protective program that restricts the expansion of potentially pre-malignant cells through the p53 and p16^INK4a^ pathways, thereby preserving tissue homeostasis and serving as a barrier against tumorigenesis [4,5]. Although chemotherapy and radiotherapy induce senescence in cancer cells to suppress their proliferation, the subsequent accumulation of these senescent tumor cells may, paradoxically, accelerate tumor progression [6]. The delay in eliminating senescent cells causes an accumulation of their senescence-associated secretory phenotype (SASP), a complex blend of cytokines, growth factors, and proteolytic enzymes that paradoxically encourages chronic inflammation [7]. The “senescence–inflammation” vicious cycle perpetuates the propagation of cellular senescence, remodels the tumor microenvironment (TME), and ultimately establishes a niche that facilitates the expansion and survival of malignant clones [8,9]. Similar TME remodeling effects have been documented in colorectal cancer [10]. Importantly, the SASP plays a role in attracting immune cells like macrophages, neutrophils, and myeloid-derived suppressor cells (MDSCs) into the TME. These invading cells also release pro-inflammatory substances like interleukin-6 (IL-6) and tumor necrosis factor-alpha (TNF-α), which in turn promote tumor growth, metastasis, and therapy resistance [11]. Concurrently, the sustained antigenic stimulation and metabolic stress triggered by SASP drive cytotoxic T cells to overexpress inhibitory receptors like programmed death-1 (PD-1) and T cell immunoglobulin mucin-3. This triggers simultaneous loss of cytokine secretion and cytotoxic function, ultimately forming a SASP-immunosuppression-adaptive immune exhaustion cascade that establishes a stable immune escape microenvironment [12,13]. Therefore, a promising therapeutic approach involves combining senescence intervention—either via senolysis or SASP signal blockade—with immune microenvironment remodeling. This integrated strategy aims to disrupt the “senescence–tumor” vicious cycle, thereby potentially reversing the immunosuppressive TME [14].

Colon adenocarcinoma (COAD) represents the most prevalent pathological type of colorectal cancer [15]. Herein, we present a quantifiable cellular senescence risk model using a core gene set as a molecular scale. From the perspective of senescence-immune crosstalk, our findings offer novel biomarkers and synergistic combination strategies for COAD immunotherapy.

## 2. Materials and Methods

### 2.1. Data Acquisition and Cohort Selection Criteria

Transcriptomic and clinical data from colorectal cancer patients, including samples from both tumor and normal tissues, were collected from the Cancer Genome Atlas (TCGA, https://portal.gdc.cancer.gov accessed on 10 May 2025) and Gene Expression Omnibus (GEO, http://www.ncbi.nlm.nih.gov/geo accessed on 10 May 2025) databases. After the screening, 448 COAD patients possessing both mRNA expression data alongside clinical details were picked from the TCGA cohort for the training batch. Gene expression levels from the TCGA cohort, originally quantified as FPKM (Fragments Per Kilobase of exon model per Million mapped fragments), were converted to TPM (Transcripts Per Million) to ensure comparability across samples for subsequent analysis. Differential expression analysis used a threshold of |log_2_FC| being over 0.585 (corresponding to a linear fold change > 1.5) and a false discovery rate (FDR) of less than 0.05. Additionally, two independent validation cohorts (GSE40967 and GSE12945) of colorectal cancer from the GEO database were included. The characteristics of the cohorts from TCGA-COAD, GSE40967, and GSE12945 were outlined in Supplementary Table S1.

### 2.2. Creation of Features Associated with Cellular Senescence

From the CellAge database, genes connected to cellular senescence were acquired (https://genomics.senescence.info/cells/ accessed on 10 May 2025). A univariate Cox proportional hazards analysis was conducted to identify genes with significant associations to overall survival (OS). OS was defined as the time interval from the date of initial pathological diagnosis to death from any cause. The applicability and consistency of this definition across the TCGA and GEO datasets were verified based on the metadata. The Venn analysis was performed with differentially expressed senescence-related genes, and genes identified as risk factors [hazard ratio (HR) > 1] were further analyzed using LASSO-Cox regression. The optimal λ value was determined through cross-validation to construct a cellular senescence-related signature (CSRS) comprising relevant genes. The CSRS score of patients were calculated based on gene expression levels, and the division was made into high-risk and low-risk groups according to the mean CSRS score. To identify independent prognostic factors for COAD patients, clinical–pathological factors like age, pathological stage, and CSRS score were combined using both univariate and multivariate methods for Cox regression analysis.

### 2.3. Functional and Pathway Enrichment Analysis

We employed the DESeq2 package (v 1.48.1) (for TCGA RNA-seq data) and limma package (v 3.64.1) (for GEO microarray data) to conduct comparative analyses between tumor and adjacent normal tissues. Subsequently, we used the clusterProfiler package in R software (version 4.4.2) to perform gene enrichment analysis with the Gene Ontology (GO) and Kyoto Encyclopedia of Genes and Genomes (KEGG) databases. The Gene Set Enrichment Analysis (GSEA, v4.4.0) software was employed to perform KEGG pathway enrichment analysis on the gene expression patterns of CSRS high-risk group patients, using the weighted scoring method with FDR < 0.05 as the significance threshold.

### 2.4. Validation of Cell Senescence-Related Features

The cutoff value for stratifying patients into high- and low-risk groups was determined independently within each validation cohort by applying the mean CSRS of that specific cohort. The creation of risk curves, survival status dot plots, and gene expression heatmaps was accomplished with the pheatmap package in R software (v 4.4.2). The survival and survminer packages were used to examine the prognostic value of CSRS score in COAD patients, with Kaplan–Meier survival curves visualizing differences across risk groups. The timeROC package (v 0.4) was utilized for ROC curve plotting. Immunophenotype scores (IPS) were derived from The Cancer Immunome Data from Atlas (TCIA, https://tcia.at accessed on 15 May 2025), and violin plots were generated using the ggpubr package (v 0.6.1).

### 2.5. Association of CSRS Score with Immune Cell Infiltration

We utilized the CIBERSORT-ABS, MCPCOUNTER and TIMER algorithms to assess immune cell infiltration. By employing R software (v 4.4.2) packages including reshape2 (v 1.4.4), ggplot2 (v 3.5.2) and ggpubr (v 0.6.1), we explored the relationships among various genes, CSRS and immune cell infiltration, and subsequently generated heatmaps.

### 2.6. Statistical Analysis

R software (v 4.4.2) and SPSS Statistics (v 25.0) facilitated all statistical analysis and the creation of charts. The Kaplan–Meier method and log-rank test enabled survival analysis among groups, and independent prognostic factors were analyzed using univariate and multivariate Cox regression. ROC curves were employed to evaluate model predictive performance. Spearman correlation analysis was used to assess correlations. All statistical tests were two-sided, with *p* < 0.05 indicating statistical significance.

## 3. Results

### 3.1. Development of a Prognostic Scoring Model for Cell Senescence

**Figure 1:**
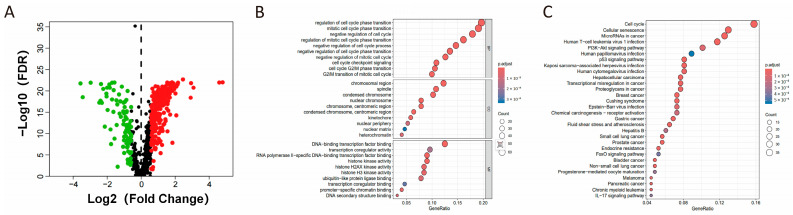
Analysis of senescence-related gene expression in patients with COAD. (**A**) Volcano plot depicting genes with differential expression associated with senescence. Red and green nodes indicate upregulated and downregulated genes, respectively. (**B**) GO and (**C**) KEGG enrichment analysis of differentially expressed senescence-associated genes.

To thoroughly examine the expression patterns of cell senescence-related genes in colorectal cancer, we studied tumor and normal tissues from the TCGA-COAD cohort, building upon previous research that utilized the TCGA database to screen and validate colorectal cancer-related genes. In our study, we leveraged the CellAge database to analyze 866 genes associated with cellular senescence and identify 346 differentially expressed genes (DEGs) in patients with COAD (Supplemental Table S2). Of these, 256 genes exhibited an upregulation trend, while 90 showed downregulation, as depicted in Figure 1A. Through the application of GO and KEGG pathway analysis, the DEGs were found to be notably enriched in pathways related to cell cycle regulation, cellular senescence, and inflammatory responses (Figure 1B,C). This enrichment is in line with the understanding that cell cycle dysregulation contributes to cellular aging and the onset of age-related diseases [16].

**Figure 2:**
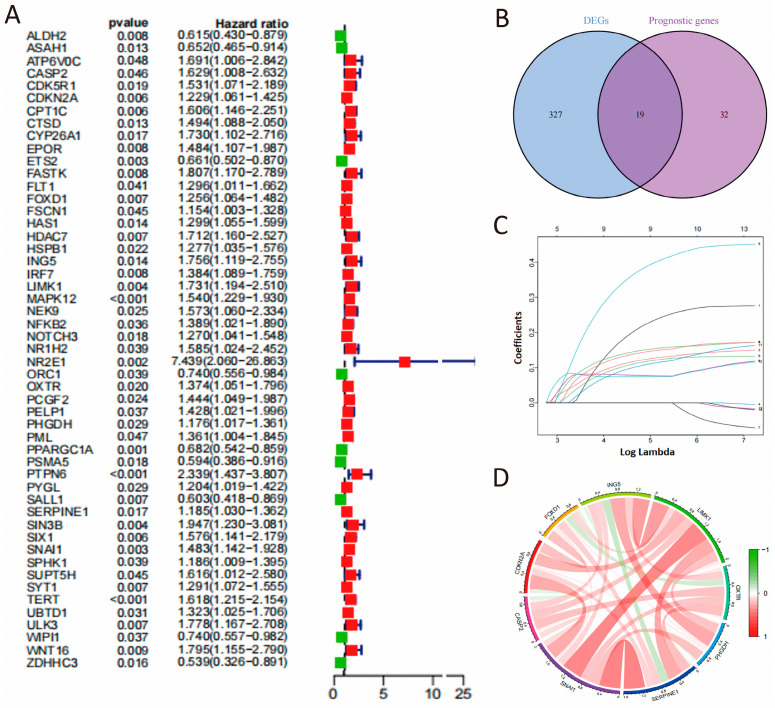
Development of the CSRS model. (**A**) The screening of prognostic genes. (**B**) Venn analysis of cell senescence-associated differentially expressed genes and prognostic genes. (**C**) LASSO coefficients of 9 candidate genes. (**D**) Correlation network of 9 candidate genes.

Subsequently, we applied univariate Cox proportional hazards regression analysis to identify genes connected to OS outcomes, finding 51 genes with a significant link to OS (*p* < 0.05, Figure 2A). Among these, 19 genes overlapped with differentially expressed senescence-related genes (Figure 2B). Ultimately, 14 genes (CASP2, CDKN2A, FOXD1, FSCN1, ING5, LIMK1, NOTCH3, OXTR, PHGDH, PML, SERPINE1, SNAI1, SPHK1, and UBTD1) were identified as risk factors (HR > 1) and all showed upregulated expression in COAD.

A 9-gene signature was then identified as optimal for COAD survival prediction through LASSO-Cox regression analysis, validated by λ values from cross-validation (Figure 2C). These genes exhibited strong positive correlations (Figure 2D). A cell senescence-related prognostic formula was developed as below: CSRS = (CASP2 × 0.2098) + (CDKN2A × 0.1196) + (FOXD1 × 0.1472) + (ING5 × 0.3723) + (OXTR × 0.0786) + (PHGDH × 0.1408) + (SERPINE1 × 0.1127) + (SNAI1 × 0.1034) + (LIMK1 × 0.0747). Patients were sorted into high-risk and low-risk categories using the CSRS score, based on the Mean value (Supplemental Table S3). Additionally, we conducted an extra analysis by directly applying LASSO to all senescence-related DEGs with *p*.adj < 0.05 and |log_2_FC| > 0.585. The final models shared core genes (e.g., CASP2, FOXD1, PHGDH, SERPINE1) with our original CSRS, and the new risk scores (risk score 1) were highly correlated with the original CSRS (r = 0.606, *p* < 0.0001), demonstrating stable prognostic predictive power in the validation sets (Supplementary Figure S1 and Supplementary Table S4). We also reran the LASSO regression starting using all genes that were significantly associated with OS (*p* < 0.05). The new risk scores (risk score 2) were also highly correlated with the original CSRS (r = 0.604, *p* < 0.0001, Supplementary Figure S1 and Supplementary Table S5).

### 3.2. Validation of the Cell Senescence-Related Prognostic Model

**Figure 3:**
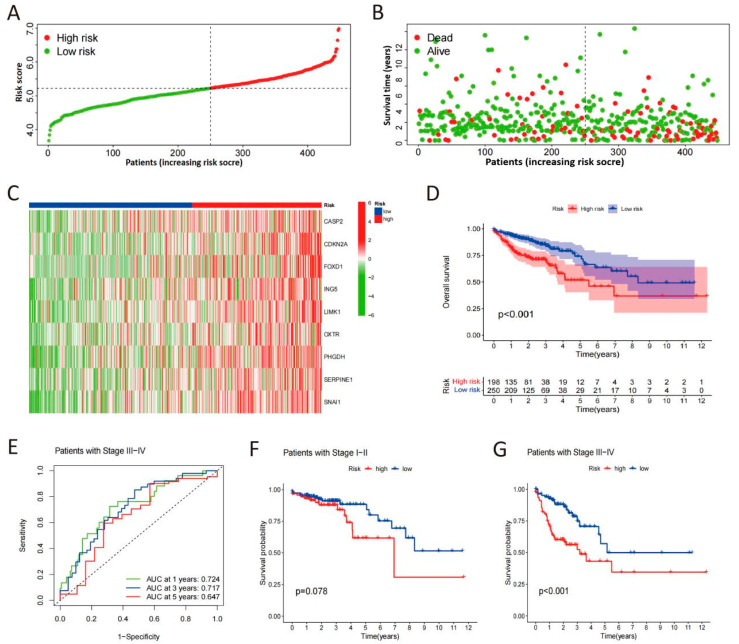
Validation of the CSRS model. (**A**) Risk score distribution of COAD patients in low- and high-risk groups. (**B**) Survival analysis of COAD patients in low- and high-risk groups. (**C**) Heat maps of expression profiles for 9 candidate genes in high- and low-risk groups. (**D**) Survival curves of COAD patients determined by risk scores. (**E**) Time-dependent ROC curves in advanced stages of COAD patients. (**F**,**G**) Survival curves in early and advanced stages of COAD patients.

The CSRS score distribution curve, survival status scatter plot, and heat maps of selected gene expression profiles for high- and low-risk groups are shown in Figure 3A–C. The Kaplan–Meier survival analysis demonstrated a significantly reduced OS in patients at high risk compared to those at low risk (Figure 3D). In advanced stages of COAD patients, time-dependent ROC analysis of the apparent performance showed that the area under the curve (AUC) for 1-year, 3-year, and 5-year OS was 0.724, 0.717, and 0.647, respectively, reflecting the predictive accuracy over these time intervals (Figure 3E). Our analysis indicates that high-risk patients, as defined by our scoring system, have a significantly reduced OS compared to low-risk patients in advanced stages of the disease (Figure 3F,G). These indicate that higher CSRS scores correlate with greater likelihood of malignant progression in COAD.

### 3.3. The Value of CSRS Model in Prognosis and Immunotherapy

**Figure 4:**
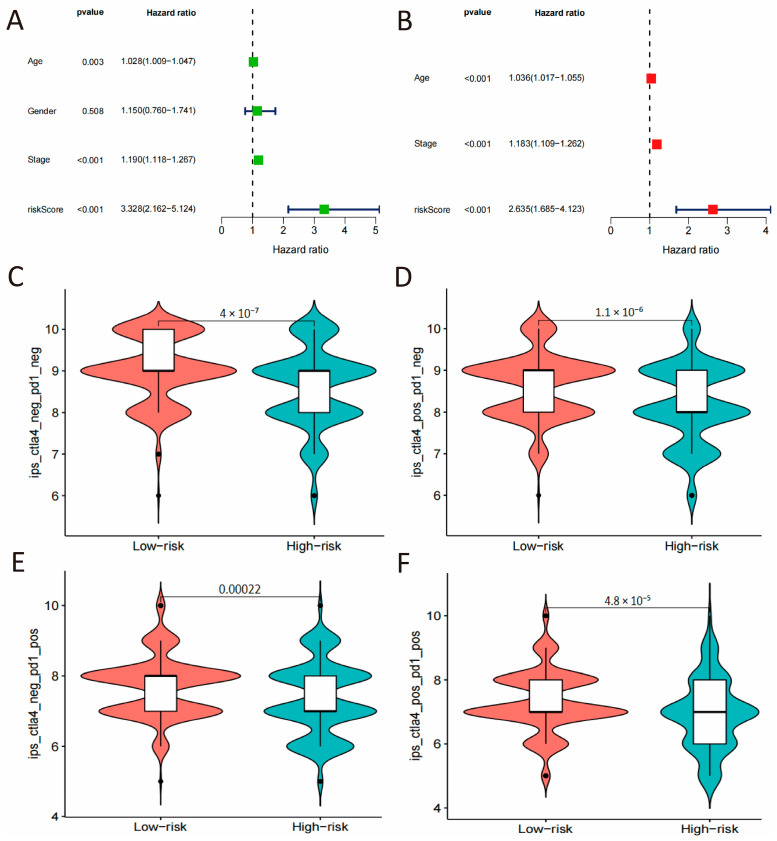
Independent prognostic validation and immunotherapeutic potential. (**A**,**B**) Univariate and multivariate Cox regression analysis of the risk score and clinicopathological parameters. (**C**–**F**) Comparisons of IPS between high- and low-risk groups under different immunotherapeutic scenarios.

In order to establish the CSRS-based risk score as an independent prognostic factor for COAD, we used Cox regression analysis in the TCGA cohort, adjusting for known clinicopathological variables, including age, sex, and pathological stage. The univariate analysis revealed that age (HR: 1.028, 95% CI: 1.009–1.047, *p* = 0.003), pathological stage (HR: 1.190,95% CI: 1.118–1.267, *p* < 0.001) and the risk score (HR: 3.328, 95% CI: 2.162–5.124, *p* < 0.001) all significantly influenced the OS of COAD patients. Even after adjusting for multiple variables, the risk score continued to be a significant independent predictor of apparent prognosis in this training set (HR: 2.635, 95% CI: 1.685–4.123, *p* < 0.001, Figure 4A,B).

The study further explored the potential value of the CSRS score in immunotherapy. The CSRS-high group exhibited significantly higher Immunophenotype Scores (IPS) than the CSRS-low group (*p* < 0.001), suggesting a potentially more immunogenic tumor microenvironment.

### 3.4. The Validation of CSRS Model in Two Independent Cohorts

**Figure 5:**
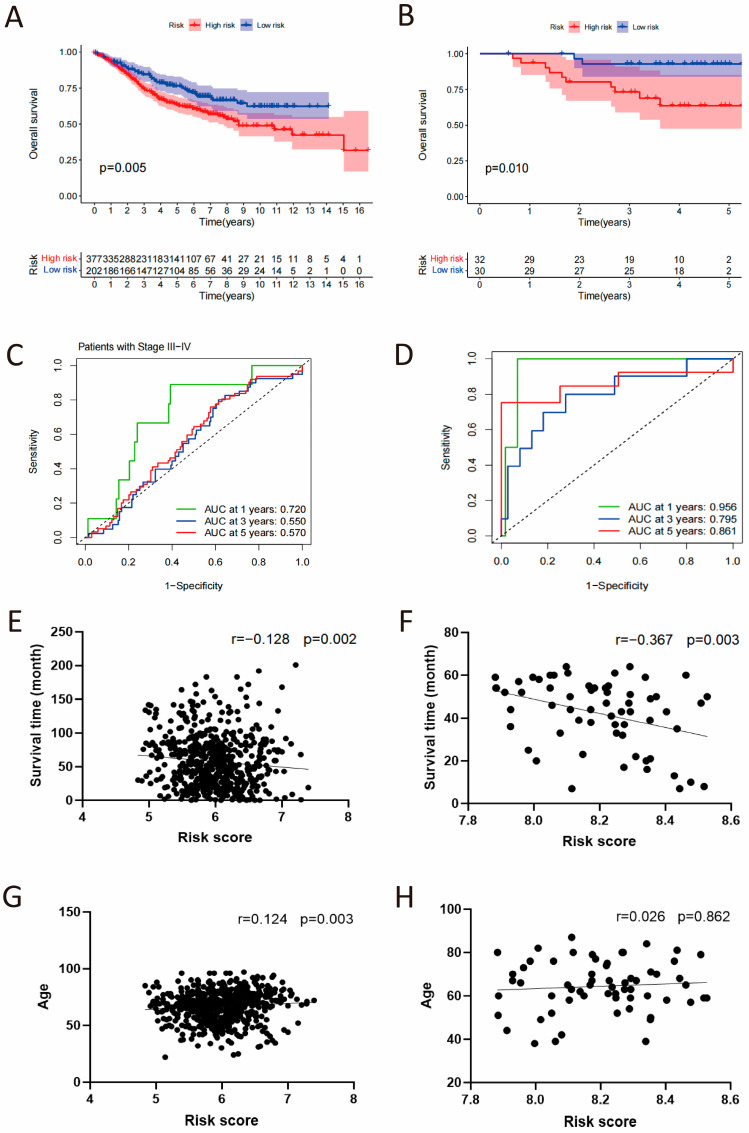
Independent validation of the CSRS model in GSE40967 and GSE12945 cohorts. (**A**,**B**) Kaplan–Meier survival analysis stratified by the CSRS. (**C**,**D**) Time-dependent ROC curves assessing the model’s predictive accuracy. (**E**–**H**) Correlation analysis of the CSRS with patient survival time and age.

To validate the prognostic function of CSRS for overall survival, this study incorporated two datasets of colorectal cancer from the GEO database. As shown in Figure 5A,B (Supplemental Tables S6 and S7), both cohorts found that patients with high-risk scores had considerably worse OS outcomes, including GSE40967 (*p* = 0.005) and GSE12945 (*p* = 0.01). In the GSE40967 cohort, the AUC values at 1, 3, and 5 years were 0.720, 0.550 and 0.570 in late-stage patients (Figure 5C), while in the GSE12945 cohort, the AUC values were 0.956, 0.795 and 0.861 (Figure 5D). Given the high AUC observed in the GSE12945 cohort, we specifically recalculated and now report the 95% confidence intervals for all AUCs using the Bootstrap method (1-year AUC: 0.957, 95% CI [0.879, 1.000], 3-year AUC: 0.813, 95% CI [0.628, 0.956] and 5-year AUC: 0.853, 95% CI [0.621, 1.000], Supplemental Figure S2). Additionally, a negative correlation was observed between CSRS risk scores and survival time (*p* = 0.002 and 0.003), whereas age showed a positive correlation with CSRS (*p* = 0.003). The findings suggest that CSRS acts as a significant prognostic indicator, especially in advanced stages of colorectal cancer patients.

### 3.5. CSRS and SASP Alterations Linked to Immune Cell Infiltration

**Figure 6:**
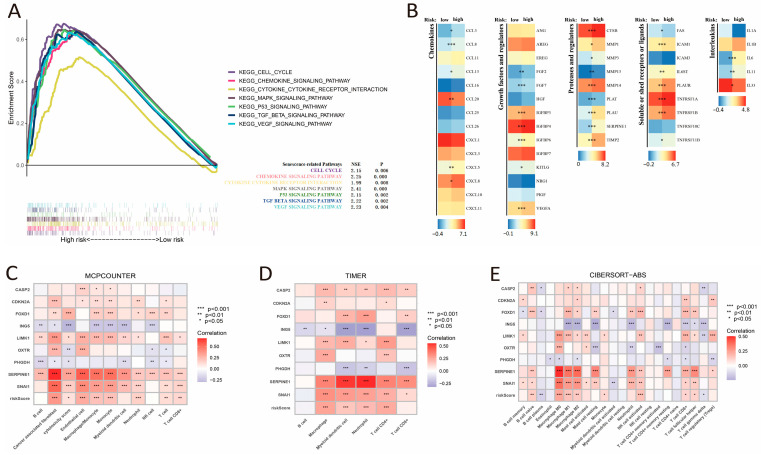
Pathway enrichment in the high-risk group, SASP expression, and the correlation between CSRS score and immune cell infiltration. (**A**) GSEA enrichment analysis of the high-risk group. (**B**) Differential expression of SASP factors between groups with high and low risk. (**C**–**E**) Correlation of risk score and genes with immune cell infiltration assessed by MCPCOUNTER, TIMER, and CIBERSORT-ABS algorithms. *, **, and *** represent *p* < 0.05, *p* < 0.01, and *p* < 0.001, respectively.

GSEA analysis identified pronounced enrichment of cellular senescence-related pathways in CSRS high-risk patients. These notably included fundamental regulatory pathways such as cell cycle, MAPK, and P53 signaling, along with the characteristic SASP components like cytokines and chemokines (Figure 6A). The high-risk group showed upregulation of multiple SASP types, including chemokines (CCL3, CCL8, CCL13, CCL20, CXCL5, and CXCL8), growth factors and regulators (FGF2, FGF7, KTTLG, VEGFAIGFBP3, IGFBP4 and IGFBP6), proteases and regulators (CTSB, MMP1, MMP3, MMP13, MMP14, PLAT, PLAU, SERPINE3, and TIMP2), soluble or shed receptors or ligands (FAS, ICAM1, ICAM3, IL6ST, PLAUR, TNFRSF1A, TNFRSF1B, and TNFRSF11B), and interleukins (IL-6, IL-11, and IL-33) (Figure 6B). As shown in immune cell infiltration analysis using CIBERSORT, MCPcounter, and TIMER algorithms, the CSRS score was negatively correlated with the infiltration of plasma cells and γδ T cells, while it demonstrated a positive correlation with the infiltration of CD4^+^ T cells, cancer-associated fibroblasts, endothelial cells, neutrophils, activated NK cells, monocytes and macrophages (Figure 6C–E).

## 4. Discussion

Initially regarded as a tumor-suppressive mechanism, cellular senescence was believed to inhibit cancer cell proliferation by halting the cell cycle and boosting immune surveillance [17]. However, recent evidence indicates that senescent tumor cells secrete SASPs, including IL-6, IL-8, along with vascular endothelial growth factor (VEGF), which can foster malignant progression in adjacent cancer cells via various mechanisms, such as recruiting MDSCs, regulatory T cells, and M2 macrophages as well as remodeling the tumor microenvironment to facilitate tumor growth and metastasis [18,19]. Consequently, aging exhibits a “double-edged sword” property in cancer therapy. This study systematically analyzed the expression profiles of senescence-associated genes in COAD patients from TCGA database, quantified intratumoral senescence activity, and constructed a prognostic model. Using a LASSO model, we constructed a CSRS model comprising nine core senescence genes (CASP2, CDKN2A, FOXD1, ING5, OXTR, PHGDH, SERPINE1, SNAI1, and LIMK1). Individuals in the low-risk category showed apparent better overall survival rates, more effective immune responses, and reduced levels of SASP factors. The CSRS score showed a positive correlation with macrophage and cancer-associated fibroblast infiltration. Notably, this model demonstrated robust survival prediction efficacy in two independent GEO cohorts.

In the CSRS model, cyclin-dependent kinase inhibitor 2A (CDKN2A), also known as p16, is a robust cellular senescence marker that has been identified with high expression in colorectal cancer tissues [20]. Our present study demonstrated that CDKN2A expression was positively correlated with the infiltration levels of CD4^+^ T cells, macrophages, and cancer-associated fibroblasts, suggesting its apparent potential role in promoting tumor progression through remodeling the immune microenvironment. Another investigation highlighted a significant positive correlation between CDKN2A expression and both immune cell infiltration and overall survival in COAD [21]. Furthermore, experimental study has demonstrated that baicalin prevents colorectal cancer by inhibiting CDKN2A, suggesting that targeting senescence markers may suppress tumor development [22]. Taken together, these data position CDKN2A as a candidate biomarker for gauging outcome in COAD. Research indicates that during radiotherapy or oxaliplatin treatment, senescent colorectal cancer cells secrete extracellular vesicles enriched with SERPINE1, which in turn binds to cytoplasmic NF-κB p65 in adjacent tumor cells, promotes its nuclear translocation, and ultimately accelerates tumor progression [23]. Beyond its association with poor survival and metastasis, SERPINE1 drives colon cancer progression through the Notch signaling pathway [24]. Moreover, its expression is upregulated by LINC02257, which sequesters tumor-suppressive miR-1273g-3p to relieve post-transcriptional repression of SERPINE1, thereby enhancing tumor cell proliferation and metastasis [25]. These results together with our study highlight SERPINE1 as a promising novel therapeutic target managing aging-associated colorectal cancer progression. Forkhead box protein D1 (FOXD1) expression in colon cancer tissues is closely correlated with tumor size, differentiation grade, depth of invasion, lymph node metastasis, and TNM stage, and its high expression often predicts poor OS outcomes [26,27]. Mechanistically, FOXD1 promotes tumor cell stemness and chemoresistance by directly binding to β-catenin and enhancing its nuclear localization [28]. Our findings further confirm that FOXD1 expression in COAD tissues exceeds normal levels and shows significant correlation with multiple immune cell infiltrations, indicating its potential as a biomarker for predicting COAD prognosis and immune microenvironment status. Inhibitor of growth protein 5 (ING5), another core component of the CSRS model, acts as a transcriptional co-activator, assisting p53 in enhancing p21 promoter activity and inducing p21 expression [29]. In the DNA damage response, ING5 acts as a cofactor for Tip60, acetylating p53 at K120, thereby promoting p53-mediated apoptosis [30]. Further studies have demonstrated that ING5 exhibits nuclear-cytoplasmic dual expression in colorectal cancer, with a positive rate significantly higher than that in hepatocellular carcinoma and other cancers [31]. Of particular importance in our prognostic model, qRT-PCR-based studies investigating ING5 expression in colorectal cancer have shown that ING5 accounts for 37.23% of the weight among the nine core genes—the highest proportion—underlining its expression level as a crucial determinant of survival outcomes in individuals with colorectal cancer. Oxytocin receptor (OXTR), another key gene in the CSRS model, is closely linked to COAD progression. The upregulation of OXTR mRNA is associated with the development and distant metastasis of COAD and predicts a poor prognosis [32]. Additionally, OXTR expression significantly correlates with the infiltration levels of type 2 helper T cells, central memory CD8^+^ T cells, activated CD8^+^ T cells, and activated B cells [32]. Phosphoglycerate dehydrogenase (PHGDH), the rate-limiting enzyme of the endogenous serine synthesis pathway, is highly expressed in colon cancer and significantly correlates with shorter OS in patients, serving as an independent adverse prognostic indicator [33]. Furthermore, PHGDH expression was higher in colorectal cancer patients with metastasis or recurrence compared to those without recurrence, and was also elevated in metastatic tumor tissues relative to primary tumor tissues, indicating that PHGDH expression is associated with colorectal cancer metastasis [34]. The oleanolic acid analog LXH-3-71 functions as a novel molecular glue that covalently binds PHGDH at Cys281, promoting its ubiquitin-mediated degradation via the DDB1-CUL4 complex and thereby suppressing colorectal cancer stemness in preclinical models [35]. Furthermore, PHGDH can activate PKM2, leading to phosphorylation of histone H3T11 and thereby alleviating cellular senescence [36]. The Snail family transcriptional repressor 1 (SNAI1) is a direct target of the miR-34 family. P53 transcriptionally activates miR-34, thereby inhibiting SNAI1 expression and consequently suppressing SNAI1-driven epithelial–mesenchymal transition (EMT) and tumor cell invasion [37]. LIM domain kinase 1 (LIMK1), a novel β-catenin kinase highly expressed in colorectal cancer and associated with poor prognosis, promotes malignant progression through its interaction with STK25, which enhances cell proliferation and metastasis [38]. Experimental data demonstrate that LIMK1 and CDK5 synergistically facilitate cancer metastasis through a mechanism dependent on phosphorylation and co-targeting LIMK1 and CDK5 significantly suppresses tumor metastasis [39]. In most studies, cysteine-aspartate specific protease 2 (CASP2) is under-expressed in colorectal cancer tissues, and its downregulation is believed to facilitate tumor cell evasion of apoptosis [40]. In our cohort, elevated CASP2 mRNA in COAD patients is potentially linked to the tumor cell senescent state at sampling.

The role of cellular senescence in tumor development remains poorly understood, with insufficient evidence clarifying its link to immune infiltration and its potential influence on immune checkpoint inhibitor efficacy [41]. By performing systematic analysis of the tumor microenvironment in COAD patients, this study demonstrates that genes linked to senescence may notably affect immune cell composition and distribution, offering robust evidence to clarify senescence-immune interactions and their clinical significance. Cancer-associated fibroblasts, found in large numbers in the TME, have the potential to enhance the resistance of tumor cells to treatment [42]. Similarly, our present study demonstrated a positive correlation of CSRS with the infiltration of cancer-associated fibroblasts. Patients with higher CSRS scores exhibit immunosuppressive tumor microenvironments, but the correlation between activated NK cell infiltration and CSRS scores varies across distinct algorithms, highlighting the dual-edged nature of tumor-associated cellular senescence and necessitating for further investigations into its regulatory mechanisms during tumor progression and staging. During early tumorigenesis, the various SASPs act as a barrier against oncogenesis by recruiting immune cells to clear stressed or damaged cells, thereby inducing cell cycle arrest and death in pre-malignant cells. However, with the accumulation of SASP factors and the establishment of a chronic inflammatory state in the tumor microenvironment, SASP can paradoxically facilitate tumor growth by stimulating angiogenesis and inducing EMT, thereby facilitating tumor growth, metastasis, and therapy resistance [9]. Understanding the molecular mechanisms of SASP induction and its functional heterogeneity within the TME is critical for developing personalized cancer therapies and improving patient outcomes. Our analysis of SASP profiles in high risk CSRS groups revealed significant enrichment of pro-inflammatory cytokines (IL-11, IL-6, IL-33), growth factors (FGF, VEGFA, IGFBP), immune modulators (ICAMs, TNFSF), and matrix metalloproteinases (MMPs). These secretory factors may facilitate immune evasion and accelerate tumor progression [43,44]. Upregulated SASP factors such as IL-6, CXCL8, and VEGF recruit and activate MDSCs and regulatory T cells through IL-6-STAT3, CXCL8-CXCR2, and VEGFA-VEGFR2 axes, while simultaneously suppressing CD8^+^ T cell function [45]. It is also observed that senescent colon cancer cells can promote EMT in adjacent cells through IL-6, MMP-3, FGF, and HGF, potentially facilitating tumor metastasis [46,47]. In the present study, the link between CSRS and IPS suggests that the senescence-associated tumor microenvironment reflected by CSRS may influence immunotherapy outcomes—a hypothesis that awaits validation in future independent cohorts of ICI-treated COAD patients.

### Limitations and Future Directions

This study has several limitations that should be addressed in future research. Most importantly, the prognostic risk model presented here is based solely on computational analyses of transcriptomic data and lacks direct experimental validation. The biological roles of the identified risk genes, particularly their clinical correlations with cellular senescence features and their functional interactions with key infiltrating immune cells (e.g., tumor-associated macrophages, TAMs), remain to be experimentally confirmed. Such validation is crucial for elucidating the model’s underlying mechanisms. Additionally, the model was developed and validated exclusively within colorectal cancer cohorts. Its performance in other cancer types remains untested, leaving it ambiguous whether it targets colorectal cancer mechanisms or has a general application to various cancers. Future work should prioritize experimental studies to functionally characterize the risk genes and extend the model’s evaluation to diverse cancer types to determine its generalizability.

Single-timepoint tumor specimen data in the present study may be insufficient to systematically elucidate the regulatory mechanisms that underlie the time-dependent phenotypic remodeling by senescence-associated genes, necessitating further investigation through both in vivo and in vitro experiments. While current CDKN2A-targeted cancer therapeutic strategies are still under exploration [48], a sequential approach that induces senescence followed by the elimination of senescent cells, or optimized combinations of therapeutic strategies with senolytics and senostatics, may enhance anti-tumor efficacy [49,50]. Furthermore, future efforts aimed at identifying key SASP regulators and their downstream effectors to construct a comprehensive biomarker profile reflecting SASP activity could pave the way for personalized cancer treatment regimens and the development of commercial cancer diagnostic kits.

## 5. Conclusions

Our present study developed and validated a nine-gene CSRS score as a prognostic biomarker in COAD, providing a tool to navigate the complex, dual function of cellular senescence in cancer. In the TCGA training set, the CSRS score was linked to apparent immune cell infiltration levels and contributed to the regulation of the SASP-mediated immune microenvironment in COAD. Integrating CSRS with specific immune checkpoint inhibitors, such as those targeting PD-1/PD-L1 pathways, can refine the predictive biomarker framework, enabling more precise risk stratification and identification of effective therapeutic targets to bolster immune responses in COAD patients. Future research should prioritize the development of novel cancer therapeutics and diagnostic kits that specifically target the heterogeneous and dynamic nature of the SASP mediators. A rational therapeutic approach, entailing either sequential senescence induction and clearance or optimized combination regimens with senolytics/senostatics, may significantly potentiate anti-tumor efficacy.

## Data Availability

The original contributions presented in this study are included in the article/supplementary material. Further inquiries can be directed to the corresponding author.

## Supplementary Materials

The following supporting information can be downloaded at: https://www.mdpi.com/article/10.3390/cimb48010114/s1.

## Abbreviations

The following abbreviations are used in this manuscript:

SASP	senescence-associated secretory phenotype
TME	tumor microenvironment
MDSCs	myeloid-derived suppressor cells
IL-6	interleukin-6
TNF-α	tumor necrosis factor- α
PD-1	programmed death-1
COAD	colon adenocarcinoma
FDR	false discovery rate
OS	overall survival
HR	hazard ratio
CSRS	cellular senescence-related signature
KEGG	Kyoto Encyclopedia of Genes and Genomes
GO	Gene Ontology
TICA	The Cancer Immunome Atlas
IPS	immunophenotype
ROC	Receiver Operating Characteristic
AUC	area under curve
CDKN2A	cyclin-dependent kinase inhibitor 2A
SERPINE1	serpin family E member 1
FOXD1	forked box protein D1
ING5	inhibitor of growth protein 5
OXTR	oxytocin receptor
PHGDH	phosphoglycerol dehydrogenase
SNAI1	snail family transcriptional repressor 1
LIMK1	LIM domain kinase 1
CASP2	cysteinyl aspartate specific proteinase 2

## References

[1] Force L.M., Kocarnik J.M., May M.L., Bhangdia K., Crist A., Penberthy L., Pritchett N., Acheson A., Deitesfeld L., Bhoomadevi A. (2025). The global, regional, and national burden of cancer, 1990–2023, with forecasts to 2050: A systematic analysis for the Global Burden of Disease Study 2023. Lancet.

[2] National Cancer Institute. (n.d.) Cancer Stat Facts: Colon and Rectum Cancer. Surveillance, Epidemiology, and End Results (SEER) Program. https://seer.cancer.gov/statfacts/html/colorect.html.

[3] Zeineddine F.A., Zeineddine M.A., Yousef A., Gu Y., Chowdhury S., Dasari A., Huey R.W., Johnson B., Kee B., Lee M.S. (2023). Survival improvement for patients with metastatic colorectal cancer over twenty years. NPJ Precis. Oncol..

[4] Chen Z., Trotman L.C., Shaffer D., Lin H.K., Dotan Z.A., Niki M., Koutcher J.A., Scher H.I., Ludwig T., Gerald W. (2005). Crucial role of p53-dependent cellular senescence in suppression of Pten-deficient tumorigenesis. Nature.

[5] Michaloglou C., Vredeveld L.C., Soengas M.S., Denoyelle C., Kuilman T., van der Horst C.M., Majoor D.M., Shay J.W., Mooi W.J., Peeper D.S. (2005). BRAFE600-associated senescence-like cell cycle arrest of human naevi. Nature.

[6] Liu B., Peng Z., Zhang H., Zhang N., Liu Z., Xia Z., Huang S., Luo P., Cheng Q. (2025). Regulation of cellular senescence in tumor progression and therapeutic targeting: Mechanisms and pathways. Mol. Cancer.

[7] Cao L., Li K., Li Q., Tong Q., Wang Y., Huang L. (2025). The controversial role of senescence-associated secretory phenotype (SASP) in cancer therapy. Mol. Cancer.

[8] Wang L., Lankhorst L., Bernards R. (2022). Exploiting senescence for the treatment of cancer. Nat. Rev. Cancer.

[9] Cuollo L., Antonangeli F., Santoni A., Soriani A. (2020). The Senescence-Associated Secretory Phenotype (SASP) in the Challenging Future of Cancer Therapy and Age-Related Diseases. Biology.

[10] Guo Y., Ayers J.L., Carter K.T., Wang T., Maden S.K., Edmond D., Newcomb P.P., Li C., Ulrich C., Yu M. (2019). Senescence-associated tissue microenvironment promotes colon cancer formation through the secretory factor GDF15. Aging Cell.

[11] Xiong J., Dong L., Lv Q., Yin Y., Zhao J., Ke Y., Wang S., Zhang W., Wu M. (2024). Targeting senescence-associated secretory phenotypes to remodel the tumour microenvironment and modulate tumour outcomes. Clin. Transl. Med..

[12] Hope J.L., Spantidea P.I., Kiernan C.H., Stairiker C.J., Rijsbergen L.C., van Meurs M., Brouwers-Haspels I., Mueller Y.M., Nelson D.J., Bradley L.M. (2019). Microenvironment-Dependent Gradient of CTL Exhaustion in the AE17sOVA Murine Mesothelioma Tumor Model. Front. Immunol..

[13] Lee J.A., Park H.E., Lee D.W., Han S.W., Kim T.Y., Jeong S.Y., Park K.J., Bae J.M., Kang G.H. (2025). Immunogenomic characteristics and prognostic implications of terminally exhausted CD8^+^ T cells in colorectal cancers. Front. Immunol..

[14] Maggiorani D., Le O., Lisi V., Landais S., Moquin-Beaudry G., Lavallée V.P., Decaluwe H., Beauséjour C. (2024). Senescence drives immunotherapy resistance by inducing an immunosuppressive tumor microenvironment. Nat. Commun..

[15] Thrumurthy S.G., Thrumurthy S.S., Gilbert C.E., Ross P., Haji A. (2016). Colorectal adenocarcinoma: Risks, prevention and diagnosis. BMJ.

[16] He S., Sharpless N.E. (2017). Senescence in Health and Disease. Cell.

[17] Park S.S., Choi Y.W., Kim J.H., Kim H.S., Park T.J. (2021). Senescent tumor cells: An overlooked adversary in the battle against cancer. Exp. Mol. Med..

[18] Zhang J.W., Zhang D., Yu B.P. (2021). Senescent cells in cancer therapy: Why and how to remove them. Cancer Lett..

[19] Yamagishi R., Kamachi F., Nakamura M., Yamazaki S., Kamiya T., Takasugi M., Cheng Y., Nonaka Y., Yukawa-Muto Y., Thuy L.T.T. (2022). Gasdermin D-mediated release of IL-33 from senescent hepatic stellate cells promotes obesity-associated hepatocellular carcinoma. Sci. Immunol..

[20] Al-Ahwal M., Gomaa W., Emam E., Qari Y., Buhmeida A., Radwi S., Al-Maghrabi B., Al-Qahtani M., Al-Maghrabi J. (2016). p16 protein is upregulated in a stepwise fashion in colorectal adenoma and colorectal carcinoma. Saudi J. Gastroenterol..

[21] Gu R., Li S., Yu B., Gu J., Guan B., Wu H. (2025). Increased CDKN2A expression correlates with resistance to platinum-based therapy and decreased infiltration of B lymphocytes in colon adenocarcinoma. Funct. Integr. Genom..

[22] Li G.G., Chu X.F., Xing Y.M., Xue X., Ihtisham B., Liang X.F., Xu J.X., Mi Y., Zheng P.Y. (2024). Baicalin Prevents Colon Cancer by Suppressing CDKN2A Protein Expression. Chin. J. Integr. Med..

[23] Zhang D., Zhang J.W., Xu H., Chen X., Gao Y., Jiang H.G., Wang Y., Wu H., Yang L., Wang W.B. (2024). Therapy-induced senescent tumor cell-derived extracellular vesicles promote colorectal cancer progression through SERPINE1-mediated NF-κB p65 nuclear translocation. Mol. Cancer.

[24] Su X., Wang X., Lai J., Mao S., Li H. (2024). Unraveling a novel hippo-associated immunological prognostic signature: The contribution of SERPINE1 in facilitating colorectal cancer progression via the notch signaling pathway. Genomics.

[25] Park M.S., Jeong S.D., Shin C.H., Cha S., Yu A., Kim E.J., Gorospe M., Cho Y.B., Won H.H., Kim H.H. (2024). LINC02257 regulates malignant phenotypes of colorectal cancer via interacting with miR-1273g-3p and YB1. Cell Death Dis..

[26] Zong Y., Miao Y., Li W., Zheng M., Xu Z., Gao H., Feng W., Xu Z., Zhao J., Shen L. (2022). Combination of FOXD1 and Plk2: A novel biomarker for predicting unfavourable prognosis of colorectal cancer. J. Cell. Mol. Med..

[27] Pan F., Li M., Chen W. (2018). FOXD1 predicts prognosis of colorectal cancer patients and promotes colorectal cancer progression via the ERK 1/2 pathway. Am. J. Transl. Res..

[28] Feng W.Q., Zhang Y.C., Gao H., Li W.C., Miao Y.M., Xu Z.F., Xu Z.Q., Zhao J.K., Zheng M.H., Zong Y.P. (2023). FOXD1 promotes chemotherapy resistance by enhancing cell stemness in colorectal cancer through β-catenin nuclear localization. Oncol. Rep..

[29] Shiseki M., Nagashima M., Pedeux R.M., Kitahama-Shiseki M., Miura K., Okamura S., Onogi H., Higashimoto Y., Appella E., Yokota J. (2003). p29ING4 and p28ING5 bind to p53 and p300, and enhance p53 activity. Cancer Res..

[30] Liu N., Wang J., Wang J., Wang R., Liu Z., Yu Y., Lu H. (2013). ING5 is a Tip60 cofactor that acetylates p53 in response to DNA damage. Cancer Res..

[31] Yang X.F., Shen D.F., Zhao S., Ren T.R., Gao Y., Shi S., Wu J.C., Sun H.Z., Zheng H.C. (2019). Expression pattern and level of ING5 protein in normal and cancer tissues. Oncol. Lett..

[32] Sun J., Xu Z., Mao Y., Zhang T., Qin Y., Hua D. (2021). Prognostic role of oxytocin receptor in colon adenocarcinoma. Open Med..

[33] Yoon S., Kim J.G., Seo A.N., Park S.Y., Kim H.J., Park J.S., Choi G.S., Jeong J.Y., do Jun Y., Yoon G.S. (2015). Clinical Implication of Serine Metabolism-Associated Enzymes in Colon Cancer. Oncology.

[34] Zhang Y., Yu H., Zhang J., Gao H., Wang S., Li S., Wei P., Liang J., Yu G., Wang X. (2021). Cul4A-DDB1-mediated monoubiquitination of phosphoglycerate dehydrogenase promotes colorectal cancer metastasis via increased S-adenosylmethionine. J. Clin. Investig..

[35] Huang Z., Zhang K., Jiang Y., Wang M., Li M., Guo Y., Gao R., Li N., Wang C., Chen J. (2024). Molecular glue triggers degradation of PHGDH by enhancing the interaction between DDB1 and PHGDH. Acta Pharm. Sin. B.

[36] Wu Y., Tang L., Huang H., Yu Q., Hu B., Wang G., Ge F., Yin T., Li S., Yu X. (2023). Phosphoglycerate dehydrogenase activates PKM2 to phosphorylate histone H3T11 and attenuate cellular senescence. Nat. Commun..

[37] Kim N.H., Kim H.S., Li X.Y., Lee I., Choi H.S., Kang S.E., Cha S.Y., Ryu J.K., Yoon D., Fearon E.R. (2011). A p53/miRNA-34 axis regulates Snail1-dependent cancer cell epithelial-mesenchymal transition. J. Cell Biol..

[38] Sun X., Li S., Lin H. (2022). LIMK1 Interacts with STK25 to Regulate EMT and Promote the Proliferation and Metastasis of Colorectal Cancer. J. Oncol..

[39] Li S.J., Liang Z.R., Liu Z.C., Luo X.P., Li J.Y., Yu X.M., Huang X.Z., He Y., Xu T.Y., Xu J.J. (2025). LIMK1 as a Novel Kinase of β-Catenin Promotes Esophageal Cancer Metastasis by Cooperating With CDK5. Adv. Sci..

[40] Tang L., Shen H., Li X., Li Z., Liu Z., Xu J., Ma S., Zhao X., Bai X., Li M. (2016). MiR-125a-5p decreases after long non-coding RNA HOTAIR knockdown to promote cancer cell apoptosis by releasing caspase 2. Cell Death Dis..

[41] Wang B., Kohli J., Demaria M. (2020). Senescent Cells in Cancer Therapy: Friends or Foes?. Trends Cancer.

[42] Meng J., Li Y., Wan C., Sun Y., Dai X., Huang J., Hu Y., Gao Y., Wu B., Zhang Z. (2021). Targeting senescence-like fibroblasts radiosensitizes non-small cell lung cancer and reduces radiation-induced pulmonary fibrosis. JCI Insight.

[43] Basisty N., Kale A., Jeon O.H., Kuehnemann C., Payne T., Rao C., Holtz A., Shah S., Sharma V., Ferrucci L. (2020). A proteomic atlas of senescence-associated secretomes for aging biomarker development. PLoS Biol..

[44] Lau L., David G. (2019). Pro- and anti-tumorigenic functions of the senescence-associated secretory phenotype. Expert Opin. Ther. Targets.

[45] Aliazis K., Christofides A., Shah R., Yeo Y.Y., Jiang S., Charest A., Boussiotis V.A. (2025). The tumor microenvironment’s role in the response to immune checkpoint blockade. Nat. Cancer.

[46] Wang N., Fang Y., Hou Y., Cheng D., Dressler E.V., Wang H., Wang J., Wang G., Li Y., Liu H. (2024). Senescent cells promote breast cancer cells motility by secreting GM-CSF and bFGF that activate the JNK signaling pathway. Cell Commun. Signal..

[47] Dalmasso G., Cougnoux A., Faïs T., Bonnin V., Mottet-Auselo B., Nguyen H.T., Sauvanet P., Barnich N., Jary M., Pezet D. (2024). Colibactin-producing Escherichia coli enhance resistance to chemotherapeutic drugs by promoting epithelial to mesenchymal transition and cancer stem cell emergence. Gut Microbes.

[48] Wang S., Lai J.C., Li Y., Tang C., Lu J., Han M., Ye X., Jia L., Cui W., Yang J. (2025). Loss of CDKN2A Enhances the Efficacy of Immunotherapy in EGFR-Mutant Non-Small Cell Lung Cancer. Cancer Res..

[49] Short S., Fielder E., Miwa S., von Zglinicki T. (2019). Senolytics and senostatics as adjuvant tumour therapy. EBioMedicine.

[50] Assouline B., Kahn R., Hodali L., Condiotti R., Engel Y., Elyada E., Mordechai-Heyn T., Pitarresi J.R., Atias D., Steinberg E. (2024). Senescent cancer-associated fibroblasts in pancreatic adenocarcinoma restrict CD8(+) T cell activation and limit responsiveness to immunotherapy in mice. Nat. Commun..

