# Supplementary material for: The Senescence-SASP Landscape in Colon Adenocarcinoma: Prognostic and Therapeutic Implications
Source: Curr Issues Mol Biol. 2026 Jan 21;48(1):114. doi: 10.3390/cimb48010114 (PMC12840354; doi:10.3390/cimb48010114)
Supplement: Supplementary file 1 [file cimb-48-00114-s001.zip › Supplemental Figures.pdf]

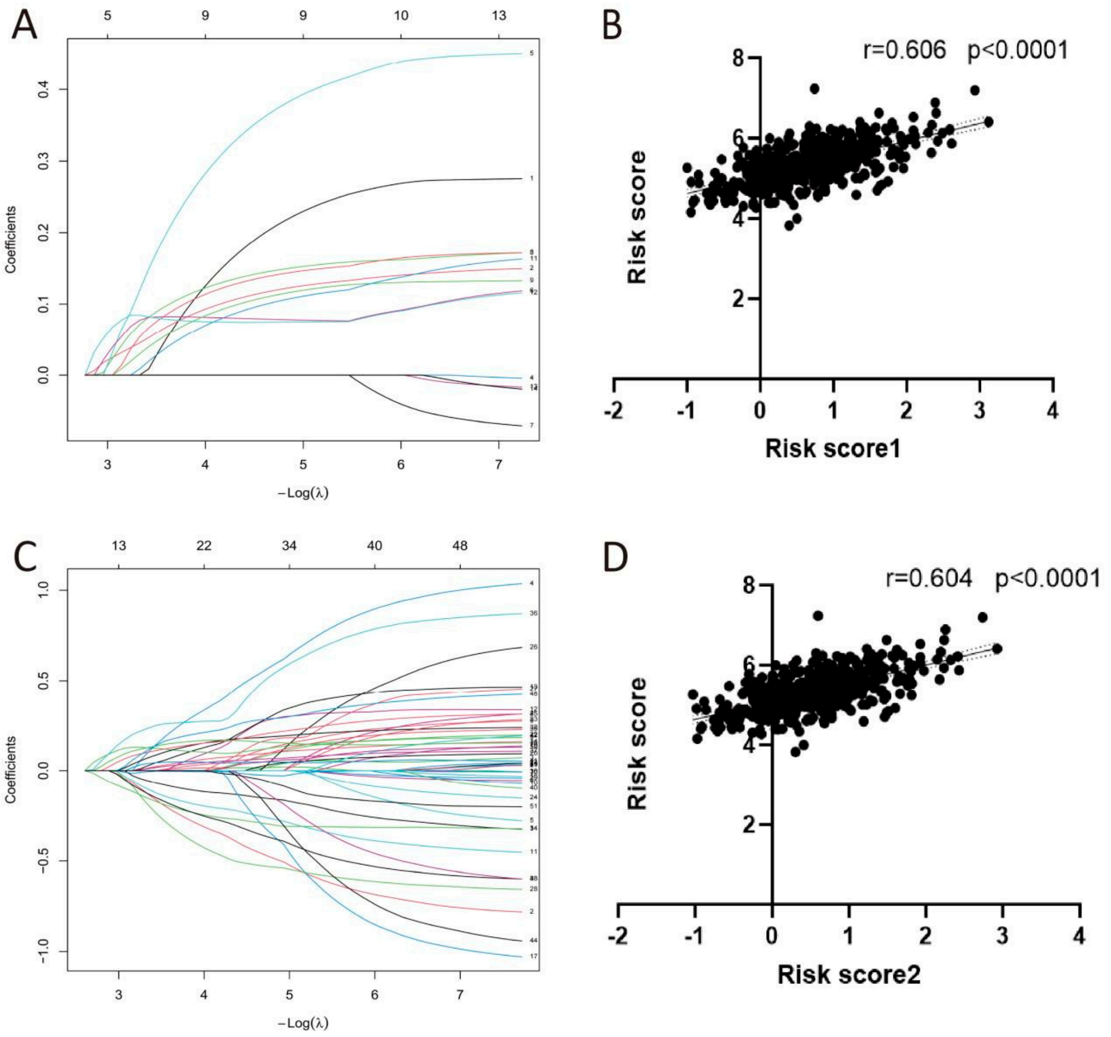

Supplementary Figure S1. Validation of the stability of the original CSRS via LASSO regression and correlation analysis. (A) LASSO coefficients of candidate genes from senescence-related DEGs (p.adj < 0.05 and |log2FC| > 0.585). (B) Correlation analysis of the original risk score with risk score 1 from (A). (C) LASSO coefficient of candidate genes from all genes significantly associated with overall survival (OS). (D) Correlation analysis of the original risk score with risk score 2 from (C).

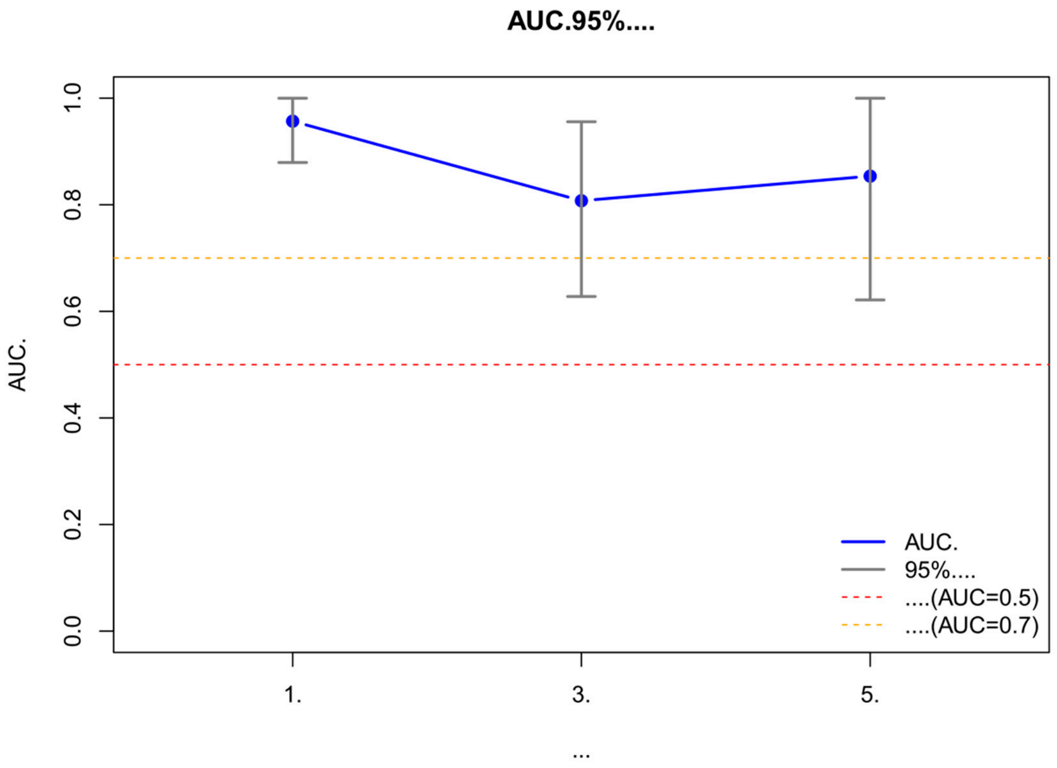

Supplementary Figure S2. The 1, 3 and 5-year AUC values and their corresponding 95% confidence intervals. The solid blue line denotes the trend of AUC values,while the gray error bars represent the 95%CIs.
